# Frailty as a predictive factor for survival after liver transplantation, especially for patients with MELD≤15—a prospective study

**DOI:** 10.1007/s00423-021-02109-9

**Published:** 2021-04-13

**Authors:** Christian G. Klein, Eugen Malamutmann, Jenny Latuske, Sefik Tagay, Nora Dörri, Martin Teufel, Andreas Paul, Arzu Oezcelik

**Affiliations:** 1 Department of General, Visceral and Transplantation Surgery, University Hospital of Essen, Hufelandstraße 55, 45147 Essen, Germany; 2 Department Psychosomatic Medicine and Psychotherapy, University of Duisburg-Essen, LVR-University Hospital, Essen, Germany

**Keywords:** Frailty index, liver transplantation, MELD score, waitlist mortality, survival

## Abstract

**Introduction:**

Frailty has been discussed as a predictor of morbidity and mortality for liver cirrhosis. The aim of our study is to evaluate the role of frailty in liver transplantation, particularly for patients with MELD scores < 15.

**Methods:**

All patients listed for liver transplantation between September 2015 and November 2018 were prospectively included in the study. Frailty was assessed by Fried’s frailty classification. Pre-, intra-, and postoperative data were prospectively recorded. Univariate and multivariate regression analyses were performed. The ethical approval of the institutional board review was obtained for the study.

**Results:**

There were 114 patients included in the study, and their median MELD score was 16. Of these, 86 patients were defined as frail (75.4%). A total of 62 patients (54.4%) underwent liver transplantation, 11 (17.7%) died postoperatively, and 24 patients (21.0%) died while on the waitlist. All postoperative mortality cases were frail, and only 3 patients (12.5%) were non-frail in the waitlist mortality group. There were 14 patients who had MELD scores of <15 (58.3%). The overall survival of non-frail patients was significantly better than that of frail patients. The multivariate regression analyses identified frailty criteria, including unintended weight loss and low hand grip strength, and platelet count and being married or living in a solid partnership were prognostic factors for survival in all patients.

**Conclusion:**

The addition of frailty assessment can be beneficial for predicting mortality after liver transplantation, especially in patients with low MELD score. Frail patients on the waitlist have significant risk for mortality even with low MELD score.

## Introduction

The most commonly used tool to determine the selection of patients for liver transplant is the model for end-stage liver disease (MELD) score. The score is composed of a logarithmic combination of serum total bilirubin levels, creatinine levels, and the international normalized ratio (INR), and it can predict 90-day mortality for most patients with cirrhosis [1]. Often, indications for liver transplant are accepted only with MELD scores > 15. However, it is also well known that in some patients, the MELD score fails to determine the patients’ general health symptoms, which include muscle wasting, malnutrition, and functional decline. These symptoms are commonly present in patients with liver cirrhosis and have a significant impact on the mortality risk pre- and post-transplant [2–7].

The MELD score is mainly dependent on serum creatinine, which can often show false-normal results in patients with liver cirrhosis due to low muscle mass and weak muscle function. This condition is defined as sarcopenia and is associated with poor overall health [8, 9]. The indication and allocation can be improved by additional factors that help to optimize the assessment of the disease severity, such as frailty and sarcopenia [3, 10, 11]. Although casually linked together, these two conditions are not the same [12].

Frailty is a complex syndrome and still not completely understood. The most extensively validated tool to measure frailty is the Fried frailty score, which consists of five components. Using this score, patients are defined as frail (score of 3–5), pre-frail (score of 1–2), and non-frail [13]. The aim of this study is to investigate the Fried frailty score for survival after liver transplant, particularly for patients with a MELD score < 15.

## Patients and methods

All patients with age >18 years who were newly listed for liver transplant between September 2015 and November 2018 were prospectively included in the study. Patients’ demographic information, etiology of the liver cirrhosis, MELD score, comorbidities, and medical history were recorded. Patients were evaluated for liver transplant following a standard evaluation program, including routine blood test, virology, duplex ultrasonography of the liver, computed tomographic angiography and portal reconstruction of the liver, pulmonary function tests, and cardiac workup. All patients included in the study were assessed at the time of enlistment in regard to frailty, laboratory values, hepatic encephalopathy, cognitive abilities, mental parameters, nursing care, and lifestyle, which included who they live with (alone, in solid partnership, with children, or with parents).

The same frailty assessment was repeated every 6 months while the patients were on the waitlist and postoperatively until the first year. Frailty was measured using the Fried frailty score, which considers unintended weight loss, low gait speed, exhaustion/weakness, and low physical activity. Gait speed was measured as the time needed to walk 5 m. Weakness/sarcopenia was evaluated by hand grip strength, which was measured using a hand dynamometer. Unintended weight loss was reported by the patient.

Patients who met at least 3 of the 5 criteria were defined as frail, while pre-frail patients were those who met 1–3 of the criteria, and non-frail patients were those who met 0–1 of the criteria. Frailty was calculated using the Johns Hopkins frailty assessment calculator. Hepatic encephalopathy was evaluated using the HEPAtonorm^TM^ analyzer. Cognitive abilities were tested using standardized cognitive tests addressing attention and memory. The Mini-Mental State test was applied to assess mental skills.

Orthotropic liver transplant was performed with a standardized technique using vena cava interposition in all patients. The intra- and postoperative data recorded included the duration of surgery, blood transfusion, cold and warm ischemic time, ICU stay, postoperative complications, hospital stay, and survival. Perioperative mortality was defined as 30-day mortality. Immunosuppressant therapy was administered after transplantation based on calcineurin inhibitors and prednisone, which were supplemented with mycophenolate mofetil. Modifications in dose or compounds were done individually according to the clinical course. All patients were followed after the transplantation according to the standard follow-up protocol. Additionally, 6 and 12 months after the liver transplant, the assessments required by the study protocol were repeated.

The waitlist mortality was defined as death before liver transplant or delisting for being too sick for liver transplant. Patients who underwent living donor liver transplantation were excluded, as were patients with acute liver failure. Patients who refused or were not able to complete all study procedures were excluded from the study. All assessments were done by the same two study personnel, who had specifically been trained in administering these study procedures. Furthermore, the assessments were done in the same order and manner for each study subject. Ethical approval from the institutional board review was obtained for the study.

Values are reported as either the median and interquartile range (IQR) or as the mean and standard deviation (SD). Continuous variables were compared using the Mann-Whitney test. Comparisons of proportions were performed using Fisher’s exact test or a chi-squared test. Overall survival curves were plotted with the Kaplan-Meier method. Because the percentage of events was less than 50%, the mean survival estimates with standard errors are reported as descriptive statistics. Univariate and multivariate hazard regression analyses were performed to identify independent prognostic factors for survival. A *p* value less than 0.05 was considered to show statistical significance. All statistical analyses were done using the software SPSS 23.0 for Windows.

## Results

A total of 114 patients were included, and their median age was 53 years (42–60 years). There were 48 female (42.1%) and 66 male (57.9%) patients. The median body mass index (BMI) was 26 (24–31), and 51 patients (44.7%) reported unintended weight loss. Hepatic encephalopathy was detected in 28 patients (24.5%). The median hand grip strength was 40.0 kg (27.8–49.0 kg), and the median gait speed was 4 seconds (3–5 seconds) per 5 m. The median MELD score was 16 (7–33).

**Fig. 1:**
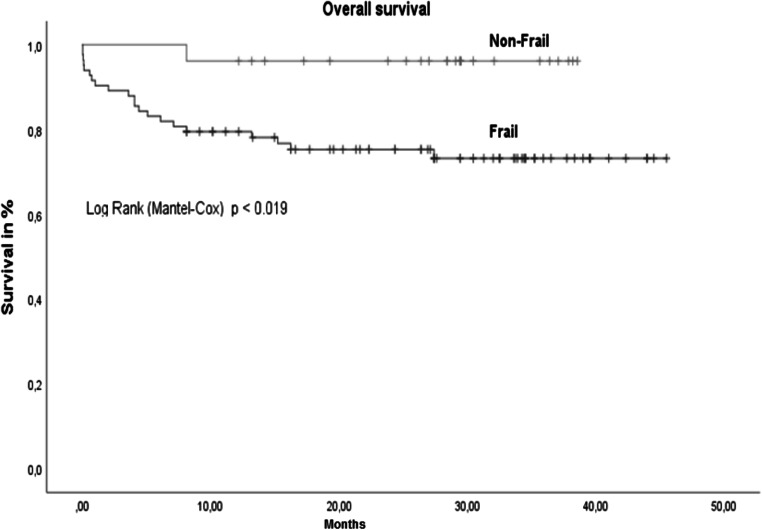
Overall survival frail vs. non-frail. *n*=114, non-frail=27 patients (23.6%) with mean survival of 25.1 (1.9) months vs. frail 87 patients (76.4%) with mean survival of 37.4 (1.1) months; *p*= 0.019

**Fig. 2:**
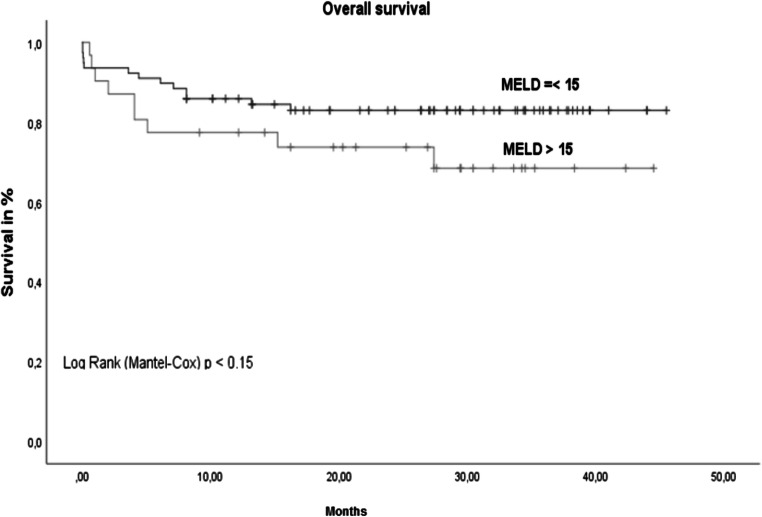
Overall survival MELD≤15 vs. MELD >15. *n*=114, MELD ≤15 =82 patients (71.9%) with mean survival of 38.6 (1.7) months vs. MELD> 15= 32 patients (28.1%) with mean survival of 33.4 (3.2) months; *p*= 0.15

A total of 29 patients (25.4%) met 3–5 of the Fried frailty criteria and were defined as frail. The criteria for pre-frail (1–2 Fried frailty criteria) were fulfilled by 58 patients (50.9%), and the remaining 27 patients (23.7%) were non-frail. The results of the statistical analyses showed no correlation between the MELD score and frailty (*p*=0.19) (Table 1). The mean overall survival of all patients was 37.2 (SD 1.2) months. The overall survival of non-frail patients was significantly better than that of frail patients (Fig. 1). No significant difference in survival was seen between patients with MELD scores <15 and above 15 (Fig. 2).

**Table 1:** Correlation between frailty and MELD score

Fried frailty score	Mean lab-MELD (standard deviation)
Non-frail (*n*=27)	12 (3.2)
Pre-frail (*n*=58)	14 (4.9)
Frail (*n*=29)	13 (4.2)

MELD= model for end-stage liver disease, *p*=0.19

A total of 62 patients (54.4%) with a median age of 54 years (44–62) underwent liver transplant using a standard technique. The median MELD score of this group was 16 (10–18). The results of the assessments for the study are shown on Table 2. There were no intraoperative complications. Blood transfusion was given to 14 patients (22.5%). The median donor risk index was 1.9 (1.4–2.1), and the median duration of ICU stay was 4 days (2–10 days). Early graft dysfunction was seen in four patients (6.4%), which was based on acute cellular rejection, and was treated successfully. Severe postoperative infection was seen in 7 patients (12.2%).

**Table 2:** Patients, underwent LT

*n*	62
Age (median)	54 (IQR 44–61)
BMI (median)	26 (IQR 23–30)
Lab-MELD (median)	16 (IQR 10–18)
Weight loss up to 5 kg (%) Weight loss more than 5 kg (%)	11 patients (17.7%) 22 patients (35.4%)
Gait speed (median)	4 seconds (IQR 3.4–5.9)
Hand grip strength (median)	40 kg (IQR 28–48.5)
Donor risk index (median)	1.9 (IQR 1.4–2.1)

BMI=body mass index, MELD=model of end-stage liver disease, kg=kilogram, IQR=interquartile range

Frailty assessment at the time of listing

**Fig. 3:**
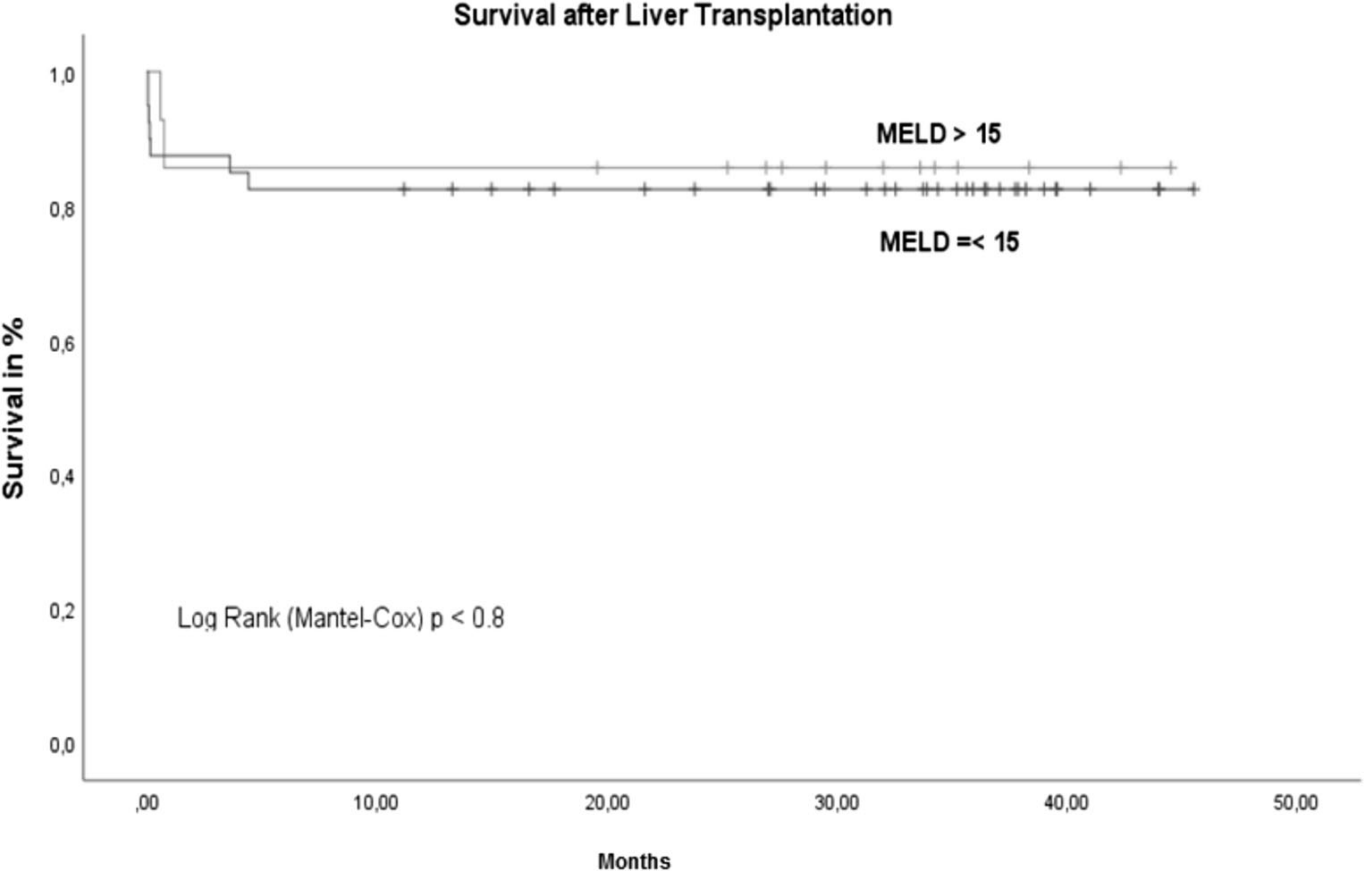
Survival after LT MELD≤15 vs. MELD >15. *n*=62, MELD ≤15= 47 patients (75.8%) with mean survival of 37.7 (2.6) months vs. MELD >15= 15 patients (24.2%) with mean survival of 38.2 (4.1) months; *p*=0.8

**Fig. 4:**
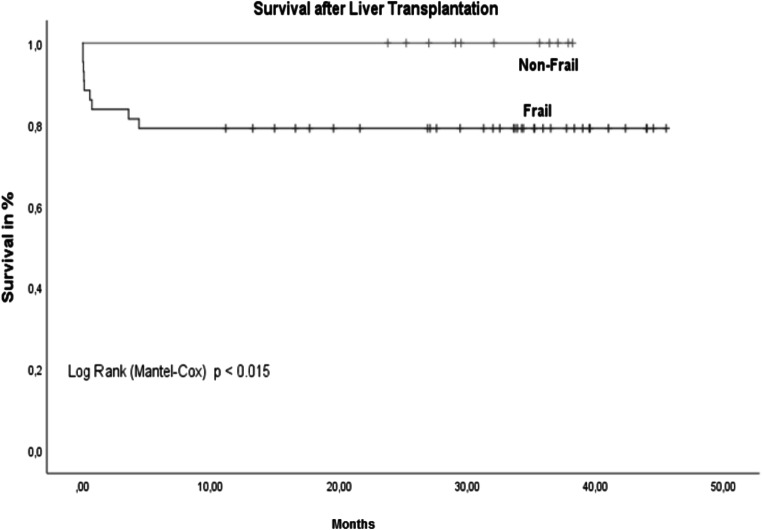
Survival after LT frail vs non-frail. *n*=62, non-frail= 13 patients (20.9%) vs. frail= 49 patients (79.1%), non-frail patients are still all alive; *p*=0.015

There were no other major surgical complications. A total of 11 patients (17.7%) died, and 7 patients (11.2%) died within the first 30 days after liver transplant. The MELD score was above 15 in only 4 of these 11 patients (36.3%), but all 11 patients (100%) fulfilled the criteria for frailty. In contrast, all non-frail patients are still alive. In the statistical comparison, frailty predicted postoperative mortality after liver transplant better than a MELD score ≤ 15 (*p*=0.003). The mean overall survival after liver transplant was 38.2 (SD 2.3) months. No significant difference was seen between patients with MELD scores <15 and above 15 (Fig. 3). The comparison of pre-frail and frail patients versus non-frail patients showed significantly better overall survival in non-frail patients (Fig. 4). There were no deaths among non-frail patients.

The waitlist mortality rate was 24% (21.1%), which was associated with a median age was 54 years (47–64 years). The results of the tests performed according to the study protocol are shown in Table 3. The median MELD score of this group was 16 (12–19). There were 10 patients (41.6%) with MELD scores above 15. Out of these 24 patients, 21 of them (87.5%) fulfilled the criteria for frailty, and only three (12.5%) were non-frail.

**Table 3:** Patients, died on the waitlist

n	24
Age (median)	54 (IQR 47–64)
BMI (median)	25 (IQR 24–39)
Lab-MELD (median)	16 (IQR 12–19)
Weight loss up to 5 kg (%) Weight loss more than 5 kg	4 patients (16.6%) 13 patients (54.1%)
Gait speed (median)	4 seconds (IQR 3.1–6.6)
Hand grip strength (median)	28.5 kg (IQR 17.8–40.5)

BMI=body mass index, MELD=model of end-stage liver disease, kg=kilogram, IQR=interquartile range

Frailty evaluation at the time of listing

The univariate analyses showed that unintended weight loss, hand grip strength, gait speed, platelet count, and being married or living in a solid partnership were significant prognostic factors for overall survival among all patients (Table 4). Factors included for the multivariate analyses are shown on Table 5. The multivariate regression analyses identified that patients with unintended weight loss, low hand-grip strength, and low platelet count had significantly higher mortality risk, while patients who were married or living in a solid partnership had significantly better overall survival (Table 5).

**Table 4:** Prognostic factors for overall survival in all patients (univariate analyses)

Variables	*x*^2^	*df*	*p* value
Encephalopathy (yes/no)	0.56	1	0.45
Weight loss (kg)	25.63	3	<0.001
Hand grip strength (kg)	5.26	1	0.022
Gait speed (seconds)	7.55	1	0.006
Platelet count (nl)	6.03	1	0.014
HEPAtonorm^TM^ analyzer (Hz)	1.75	1	0.19
Lifestyle	14.49	4	0.006

Kg=kilogram, Hz=hertz, nl=nanoliter

**Table 5:** Prognostic factors for overall survival in all patients (multivariate analyses)

Variables	SE	*Z*	*p* value	Odds ratio
Age	1.1	2.0	0.43	0.3
BMI	0.07	−1.93	0.054	0.88
MELD score	1.03	−1.45	0.07	0.82
Encephalopathy	0.78	−0.74	0.45	0.56
Weight loss (kg)	1.07	−2.41	0.016	0.08
Hand grip strength (kg)	0.03	−2.13	0.033	0.94
Gait speed (seconds)	0.42	−1.95	0.051	0.44
Platelet count (nl)	0.01	−2.12	0.034	0.99
HEPAtonorm^TM^ analyzer (Hz)	0.03	−1.25	0.210	0.97
Lifestyle				
Living alone	0.99	0.29	0.77	1.34
Living in marriage or partnership	1.41	2.60	0.009	6.20
Living with partner and children	1.83	0.13	0.893	1.28
Living with parents	1.65	0.18	0.857	1.35

Kg=kilogram, Hz=hertz, nl=nanoliter

## Discussion

Despite great efforts to raise awareness for organ donation, the gap between available organs and patients on the waitlist remains. Organ shortage makes organ allocation a challenging problem that still needs to be addressed. Ideally, the allocation should be based on the urgency of liver transplant and the expected outcome, which should be balanced. The organ allocation in most countries is mainly based on only the MELD score, which is generally accepted to be a reliable prognostic factor for 90 days waitlist mortality [1, 14]. Furthermore, a MELD score of at least 15 is required in many allocation systems as the main indication for liver transplant.

However, the clinical presentation can be extremely heterogeneous at lower MELD scores. For instance, patients with significant portal hypertension or sarcopenia with a MELD of 15 or below are clearly underrepresented by the MELD score and have a very high risk of delisting or death while on the waitlist [15]. Consequently, the MELD score alone is insufficient for optimal organ allocation. Frailty is a multidimensional evaluation characteristic and represents one of the systemic manifestations of multiple physiologic derangement and increased vulnerability to health stressors [16].

Lai et al. evaluated the influence of frailty on the waitlist mortality of liver transplant candidates. They analyzed 294 liver transplant waitlist candidates with a median age of 60 years. Frailty was defined using the Fried frailty score, and patients with a score of at least 3 were categorized as frail. Their results showed that frailty is a significant prognostic factor for waitlist mortality. One unit increase in the Fried frailty score was associated with a 50% increase risk in waitlist mortality [17]. Furthermore, patients with MELD less than 18 benefit the most from implementation of frailty criteria [18–20]. The results of our study confirm these data.

Lai et al. developed a new frailty index to predict mortality in patients with end-stage liver disease. The frailty was measured by four performance-based tests of gait speed, grip strength, chair stand, and balance, as well as five self-reported tests regarding unintentional weight loss, exhaustion, physical activity, activities of daily living, and instrumental activities of daily living. All of these factors of physical frailty except for unintentional weight loss were significantly associated with waitlist mortality in univariate Cox regression analyses. The frailty index was calculated based on these measurements, and it was shown that the combination of the MELD score and frailty index was more likely to predict waitlist mortality than either the MELD score or the frailty index alone [21].

Not only the waitlist mortality but also the mortality after liver transplant has been proven to be influenced by frailty [22]. The survival after liver transplant was significantly better in non-frail patients in our report. We may argue that the outcome of liver transplant might be improved through prehabilitation programs in a pre-transplant setting. When frailty is assessed routinely, pre-frail or frail patients could be detected early and could undergo prehabilitation processes during their time on the waitlist, which could potentially lead to faster recovery or better outcomes.

There are no established prehabilitation protocols for patients awaiting liver transplant in the literature. Williams et al. studied the feasibility of home-based exercise therapy and published their protocol, but the results are not finalized [24]. In addition, the improvement of frailty after transplantation also depends on the frailty status before transplantation [23]. Overall, more robust data are needed regarding the impact of prehabilitation programs on liver transplant outcomes. An important factor of frailty is sarcopenia. The tools to measure sarcopenia differ between studies. Here, we have chosen hand grip strength since we used the Fried frailty assessment. Another good tool is psoas muscle measurement in computed tomography, which we used in another study with good results. It is likely that sarcopenia can be improved by prehabilitation as well.

One of the shortcomings of our study is the small sample size. However, taking into account the fact that the study was performed as a prospective study, the total number of 114 patients might be acceptable. The number of patients who died while on the list or perioperatively after liver transplant was also low for statistical analysis. One strength is the high quality of the data since patients were assessed by the same team using a standardized study protocol. Another important point is that the majority of these patients had low MELD scores, which was the main focus of the study. Different tools to define frailty could be used for better comparison. The follow-up regarding frailty assessment after liver transplant was too short to draw significant conclusions for post-transplant frailty assessment. However, we are working on a follow-up study with the same patients to address this issue.

## Conclusion

The results of this study and other published data suggest that frailty among patients on the waitlist for liver transplantation should be objectively and routinely assessed. It should also be considered for organ allocation models in conjunction with the MELD score. We have shown that in patients with low MELD scores in particular, the addition of frailty assessment can be used to determine the right timing for liver transplantation. The development of concepts for prehabilitation programs for patients waiting for liver transplant seems to be the next step in improving the outcomes after liver transplant.

## Abbreviations

BMI: Body mass index
Hz: Hertz
ICU: Intensive care unit
INR: International normalized ratio
IQR: Inter quartile range
Kg: Kilogram
LT: Liver transplantation
MELD: Model for end-stage liver disease
nl: Nanoliter

## References

[1] Kamath PS, Wiesner RH, Malinchoc M, Kremers W, Therneau TM, Kosberg CL, D'Amico G, Dickson ER, Kim WR (2001). A model to predict survival in patients with end-stage liver disease. Hepatology.

[2] Montano-Loza AJ, Duarte-Rojo A, Meza-Junco J, Baracos VE, Sawyer MB, Pang JX, Beaumont C, Esfandiari N, Myers RP (2015). Inclusion of Sarcopenia Within MELD (MELD-Sarcopenia) and the Prediction of Mortality in Patients With Cirrhosis. Clin Transl Gastroenterol.

[3] Lai JC, Rahimi RS, Verna EC, Kappus MR, Dunn MA, McAdams-DeMarco M, Haugen CE, Volk ML, Duarte-Rojo A, Ganger DR (2019). Frailty Associated With Waitlist Mortality Independent of Ascites and Hepatic Encephalopathy in a Multicenter Study. Gastroenterology.

[4] Kruse JA, Thill-Baharozian MC, Carlson RW (1988). Comparison of clinical assessment with APACHE II for predicting mortality risk in patients admitted to a medical intensive care unit. JAMA.

[5] Charlson ME, Hollenberg JP, Hou J, Cooper M, Pochapin M, Pecker M (2000). Realizing the potential of clinical judgment: a real-time strategy for predicting outcomes and cost for medical inpatients. Am J Med.

[6] Rockwood K, Song X, MacKnight C, Bergman H, Hogan DB, McDowell I, Mitnitski A (2005). A global clinical measure of fitness and frailty in elderly people. CMAJ.

[7] Jasseron C, Francoz C, Antoine C, Legeai C, Durand F, Dharancy S (2019). collaborators: Impact of the new MELD-based allocation system on waiting list and post-transplant survival - a cohort analysis using the French national CRISTAL database. Transpl Int.

[8] Kaiser T, Kinny-Koster B, Bartels M, Parthaune T, Schmidt M, Thiery J (2014). Impact of different creatinine measurement methods on liver transplant allocation. PLoS One.

[9] van Vugt JLA, Buettner S, Alferink LJM, Bossche N, de Bruin RWF, Darwish Murad S, Polak WG, Metselaar HJ, IJzermans JNM (2018). Low skeletal muscle mass is associated with increased hospital costs in patients with cirrhosis listed for liver transplantation-a retrospective study. Transpl Int.

[10] Kahn J, Wagner D, Homfeld N, Muller H, Kniepeiss D, Schemmer P (2018). Both sarcopenia and frailty determine suitability of patients for liver transplantation-A systematic review and meta-analysis of the literature. Clin Transpl.

[11] Haugen CE, McAdams-DeMarco M, Holscher CM, Ying H, Gurakar AO, Garonzik-Wang J, Cameron AM, Segev DL, Lai JC (2020) Multicenter study of age, frailty, and waitlist mortality among liver transplant candidates. Ann Surg 271(6):1132–1136 10.1097/SLA.0000000000003207 PMC6639152 30672803

[12] Schopmeyer L, El Moumni M, Nieuwenhuijs-Moeke GJ, Berger SP, Bakker SJL, Pol RA (2019). Frailty has a significant influence on postoperative complications after kidney transplantation-a prospective study on short-term outcomes. Transpl Int.

[13] Fried LP, Tangen CM, Walston J, Newman AB, Hirsch C, Gottdiener J, Seeman T, Tracy R, Kop WJ, Burke G (2001). Frailty in older adults: evidence for a phenotype. J Gerontol A Biol Sci Med Sci.

[14] Wiesner R, Edwards E, Freeman R, Harper A, Kim R, Kamath P, Kremers W, Lake J, Howard T, Merion RM (2003). Model for end-stage liver disease (MELD) and allocation of donor livers. Gastroenterology.

[15] Laube R, Wang H, Park L, Heyman JK, Vidot H, Majumdar A, Strasser SI, McCaughan GW, Liu K (2018). Frailty in advanced liver disease. Liver Int.

[16] Lai JC, Sonnenday CJ, Tapper EB, Duarte-Rojo A, Dunn MA, Bernal W, Carey EJ, Dasarathy S, Kamath BM, Kappus MR (2019). Frailty in liver transplantation: An expert opinion statement from the American Society of Transplantation Liver and Intestinal Community of Practice. Am J Transplant.

[17] Lai JC, Feng S, Terrault NA, Lizaola B, Hayssen H, Covinsky K (2014). Frailty predicts waitlist mortality in liver transplant candidates. Am J Transplant.

[18] Tandon P, Ney M, Irwin I, Ma MM, Gramlich L, Bain VG, Esfandiari N, Baracos V, Montano-Loza AJ, Myers RP (2012). Severe muscle depletion in patients on the liver transplant wait list: its prevalence and independent prognostic value. Liver Transpl.

[19] Carey EJ, Steidley DE, Aqel BA, Byrne TJ, Mekeel KL, Rakela J, Vargas HE, Douglas DD (2010). Six-minute walk distance predicts mortality in liver transplant candidates. Liver Transpl.

[20] Lai JC, Dodge JL, Sen S, Covinsky K, Feng S (2016). Functional decline in patients with cirrhosis awaiting liver transplantation: Results from the functional assessment in liver transplantation (FrAILT) study. Hepatology.

[21] Lai JC, Covinsky KE, Dodge JL, Boscardin WJ, Segev DL, Roberts JP, Feng S (2017). Development of a novel frailty index to predict mortality in patients with end-stage liver disease. Hepatology.

[22] Kobashigawa J, Dadhania D, Bhorade S, Adey D, Berger J, Bhat G, Budev M, Duarte-Rojo A, Dunn M, Hall S (2019). Report from the American Society of Transplantation on frailty in solid organ transplantation. Am J Transplant.

[23] Lai JC, Segev DL, McCulloch CE, Covinsky KE, Dodge JL, Feng S (2018). Physical frailty after liver transplantation. Am J Transplant.

[24] Williams FR, Vallance A, Faulkner T, Towey J, Kyte D, Durman S, Johnson J, Holt A, Perera MT, Ferguson J (2018). Home-based exercise therapy in patients awaiting liver transplantation: protocol for an observational feasibility trial. BMJ Open.

